# Mathematical models of lignin biosynthesis

**DOI:** 10.1186/s13068-018-1028-9

**Published:** 2018-02-09

**Authors:** Mojdeh Faraji, Luis L. Fonseca, Luis Escamilla-Treviño, Jaime Barros-Rios, Nancy Engle, Zamin K. Yang, Timothy J. Tschaplinski, Richard A. Dixon, Eberhard O. Voit

**Affiliations:** 1 The Wallace H. Coulter, Department of Biomedical Engineering, Georgia Institute of Technology and Emory University, 313, Ferst Drive, Atlanta, GA 30332 USA; 2 BioEnergy Sciences Center (BESC), Oak Ridge National Lab, Oak Ridge, TN USA; 3 Department of Biological Sciences, University of North Texas, 1155 Union Circle #305220, Denton, TX 76203-5017 USA; 4 Oak Ridge National Laboratory, P.O. Box 2008, Oak Ridge, TN 37831 USA

**Keywords:** *Brachypodium distachyon*, *Medicago truncatula*, *Panicum virgatum*, Pathway analysis, *Populus trichocarpa*, Recalcitrance

## Abstract

**Background:**

Lignin is a natural polymer that is interwoven with cellulose and hemicellulose within plant cell walls. Due to this molecular arrangement, lignin is a major contributor to the recalcitrance of plant materials with respect to the extraction of sugars and their fermentation into ethanol, butanol, and other potential bioenergy crops. The lignin biosynthetic pathway is similar, but not identical in different plant species. It is in each case comprised of a moderate number of enzymatic steps, but its responses to manipulations, such as gene knock-downs, are complicated by the fact that several of the key enzymes are involved in several reaction steps. This feature poses a challenge to bioenergy production, as it renders it difficult to select the most promising combinations of genetic manipulations for the optimization of lignin composition and amount.

**Results:**

Here, we present several computational models than can aid in the analysis of data characterizing lignin biosynthesis. While minimizing technical details, we focus on the questions of what types of data are particularly useful for modeling and what genuine benefits the biofuel researcher may gain from the resulting models. We demonstrate our analysis with mathematical models for black cottonwood (*Populus trichocarpa*), alfalfa (*Medicago truncatula*), switchgrass (*Panicum virgatum*) and the grass *Brachypodium distachyon*.

**Conclusions:**

Despite commonality in pathway structure, different plant species show different regulatory features and distinct spatial and topological characteristics. The putative lignin biosynthes pathway is not able to explain the plant specific laboratory data, and the necessity of plant specific modeling should be heeded.

## Background

The recalcitrance of woody plant materials to enzymatic fermentation is the result of numerous molecular processes and features. At its core is the phenolic polymer lignin, which is interwoven with cellulose and hemicellulose, and thereby impedes access of cellulolytic enzymes, necessitating costly physico-chemical pretreatments before effective microbial fermentation can take place. With the exception of cellulose, lignin is the most abundant terrestrial biopolymer and accounts for roughly 30% of all organic carbon in the biosphere [1]. It gives a plant its structural stability, waterproofs the cell wall, thereby enabling water transport through the xylem, and protects the plant against pathogen threats. Lignin is an aromatic heteropolymer composed mainly of three types of hydroxycinnamyl alcohol monomers, namely the monolignols *p*-coumaryl alcohol, coniferyl alcohol, and sinapyl alcohol, which are commonly called H-, G-, and S-lignin, respectively.

Both the amount and composition of lignin are thought to be correlated with the hardness as well as the recalcitrance of structural plant materials. It is therefore important to the production and manipulation of bioenergy crops to understand the details of lignin synthesis and the deposition and polymerization of monolignols in the plant cell wall. In particular, the question arises whether it is possible and feasible to intervene in the phenylpropanoid pathway of lignin biosynthesis in a targeted and effective manner, for instance through gene knock-downs. The answer to this question is evidently preconditioned on detailed knowledge of this pathway and its control in situ. This knowledge in turn requires different types of biological data and, in cases where these are difficult to understand, the use of computational models that are capable of integrating small or large datasets of the same or different types and explaining observations that are sometimes unexpected.

In this article, we discuss computational models that are beneficial for explaining counterintuitive aspects of lignin biosynthesis and for making predictions regarding rational alterations in the molecular make-up of the pathway. We decided to present this material not in the form of a typical modeling paper, which would inform fellow modelers regarding all steps and technical details of model design, parameter estimation, methods of diagnostics and analysis, and interpretation of results. Instead, this paper is intended to address the practicing bioenergy scientist or engineer. It focuses on two overarching questions. First, what kinds and quantities of biological information are needed, or particularly beneficial, for setting up models of lignin synthesis and recalcitrance that have explanatory or predictive power? And second, if we succeed in constructing and implementing an effective model, what genuinely new insights might this model be able to offer? Guided by these questions, we will brush over most of the typical mathematical modeling steps and refer the reader to details in pertinent articles and reviews in the published literature.

It may surprise newcomers to the field of computational modeling that even within the limited scope of metabolic pathway modeling, the choices of mathematical formats and methods are all but unlimited. There is not “one” model that is somehow optimal, but there are many distinct options and numerous nuances. Even the representation of an enzyme catalyzed reaction can take a variety of mathematical formats, which are the result of different assumptions and focus either on molecular mechanisms or on the systemic behavior of a pathway system [2]. Studying these questions in detail, one comes to the conclusion that the selection of a model should ultimately be driven by the available data and by the scientific questions that the model is supposed to answer [3].

**Fig. 1:**
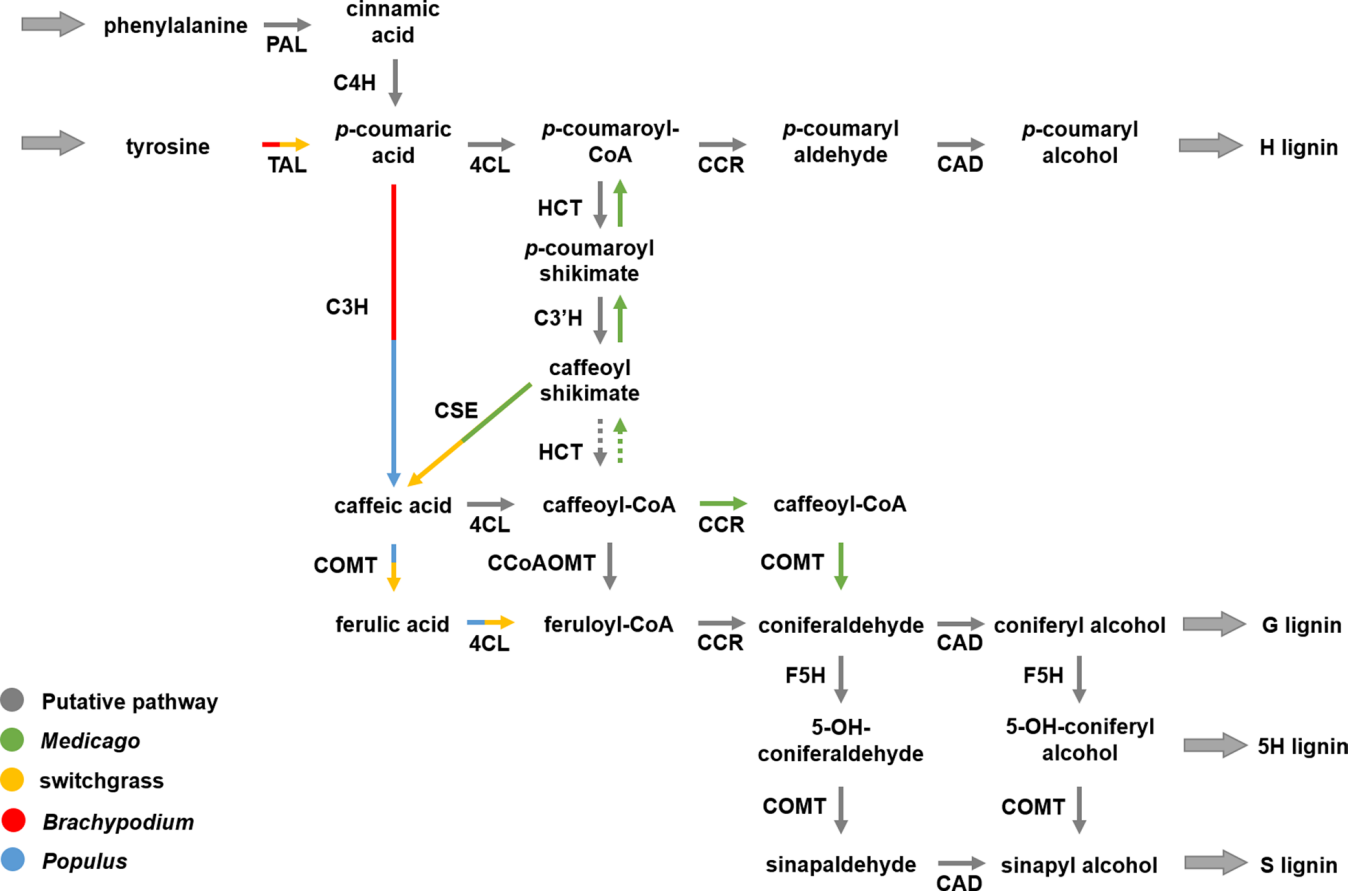
Putative lignin biosynthesis pathway with identification of species-specific reactions. Generic reactions, mainly from studies in the model dicot *Arabidopsis thaliana*, are shown in grey. Other enzymatic reactions are color coded based on the plant species where they were documented. Multicolored arrows represent reactions present in more than one species. *PAL* phenylalanine ammonia-lyase, *TAL* tyrosine ammonia-lyase, *C4H* cinnamate 4-hydroxylase, *C3H p*-coumarate 3-hydroxylase, *C3′H p*-coumaroyl shikimate 3-hydroxylase, *COMT* caffeic acid *O*-methyltransferase, *F5H* ferulate 5-hydroxylase, *4CL* 4-coumarate:CoA ligase, *HCT* hydroxycinnamoyl-CoA:shikimate hydroxycinnamoyl transferase, *CCoAOMT* caffeoyl-CoA *O*-methyltransferase, *CCR* cinnamoyl-CoA reductase, *CAD* cinnamyl alcohol dehydrogenase, *CSE* caffeoyl shikimate esterase. Interestingly, some monocots, such as *Brachypodium* and maize, do not have CSE ortholog genes. Dashed arrows are currently considered less efficient metabolic reactions in vivo

Although the structure of the lignin polymer is rather similar among different plant species, targeted experiments have revealed that the pathway of lignin biosynthesis exhibits variations among these same plant species. These variations are primarily manifest in the presence or absence of some of the involved enzymes, secondarily in different enzyme activities and substrate affinities, and third in possibly different regulatory control structures. As an illustration, Fig. 1 overlays the pathways of lignin biosynthesis in *Populus trichocarpa* (black cottonwood poplar), *Medicago sativa* (alfalfa), *Panicum virgatum* (switchgrass), and the model grass *Brachypodium distachyon*, as far as they are known or suspected today. A commonality among these species is that the pathway of lignin biosynthesis uses phenylalanine as its starting substrate; however, monocot grasses, including *B. distachyon*, and possibly *P. virgatum* as well, also use tyrosine, in addition to phenylalanine. It is presumably a biochemical necessity that most intermediates between these initial substrates and the final monolignols are by and large preserved, but the pathway systems in the species are connected in a slightly different manner through enzymatic reactions. These differences are not only of academic interest to the evolutionary biologist, but also of great significance to the biofuel researcher, because targeted interventions are almost always based on specific changes in gene expression, with concomitant alterations in fluxes through enzymatic reaction steps, such that a precise understanding of the details of the metabolic system is a prerequisite for targeted manipulations.

Predicting global effects of such manipulations on the ultimate lignin output and composition is not trivial, because the pathway utilizes the same enzymes for different reaction steps, but presumably with different substrate affinities (Fig. 1). Furthermore, the pathway is regulated, and some reactions occur in different locations of the cell and some may form functional metabolic channels. Details of the latter insights were actually derived from computational models that demonstrated that the absence of these features was inconsistent with experimental findings, as we will discuss later in this article.

## Data needs for different modeling approaches and uses of model output

### An ideal dataset

In an ideal modeling world, experimental teams would be able to measure every piece of information needed to create a comprehensive model. The data would be of high quality, obtained in situ, from the same species and from multiple organisms. Obviously, this high bar cannot often be reached, and one must ask instead what compromises are still sufficient for modeling. We discuss this issue in the following.

To design and explore a model with computational methods, one needs to choose proper functional forms for the fluxes and determine their parameters. In a true mechanistic model, the mathematical format of a flux corresponds directly to the alleged biophysical or chemical mechanism, and typical parameters may be pH and temperature, and more specifically for metabolic models, may include quantities such as *V*_max_, *K*_*M*_, *K*_cat_, or *K*_*i*_, which correspond to rates and affinities in conceptual frameworks like the Michaelis–Menten mechanism.

In an idealized modeling situation, two scenarios can lead to a full model. First, knowledge of all metabolite concentrations and of all mechanisms, including input to the system, along with a complete set of physical and kinetic parameters, measured in vivo, can quite easily be converted into a comprehensive model. However, even in this quite unrealistic case, the model would ignore the spatial distribution of processes and stochastic events, which could, for instance, be due to environmental randomness or to very low numbers of enzyme or substrate molecules. Second, knowledge of all fluxes of the system and a complete set of measured physical parameters would allow the design of the model, again with the same limitation as before. At present, neither scenario is realistic, and missing information must be obtained from other sources, such as in vitro measurements, or inferred through computational means.

At this point, many modeling approaches and methods are readily available that could create functioning models out of such data, if they were available. However, they are not, and the more important point therefore is to realign the existing modeling techniques with the realities of data acquisition in a field where some of the key metabolic intermediates are below the level of solid quantification.

As a premier example, flux balance analysis (FBA) [4] and its extensions are based on a mathematical framework that allows assessments of the distribution of fluxes within a metabolic pathway at a steady state under the assumption of an alleged objective of the cell or organism, such as maximal growth, the maximal efflux of some metabolite, or the production of a compound like lignin. FBA formulates the operation of the pathway system as a so-called “linear programing problem” that optimizes the chosen objective, while satisfying biological constraints, such as non-negativity and maximal magnitudes of fluxes.

FBA is a computationally simple, yet powerful tool that has been widely used in many contexts, including plant systems. For instance, in a plant context, Paez et al. [5] analyzed biomass synthesis in *Chlamydomonas reinhardtii* under different CO_2_ conditions, and Chang et al. [6] presented a genome-scale metabolic network model of the same organism. An interesting variation of FBA is the method of minimization of metabolic adjustment (MOMA) [7], which in a mutated organism tries to emulate a flux distribution that most closely mimics the wild type. Lee et al. [8] used MOMA to analyze data from knock-down experiments with genes associated with lignin biosynthesis in alfalfa.

While FBA and MOMA focus on the important distribution of fluxes at a steady state, dynamic modeling attempts to capture time-dependent changes in metabolites following any sort of perturbation. The hope is not only to understand short-term responses better, but also to capture regulatory features of the pathway system that are likely to become critical when the system is mutated. Expressed differently, FBA by and large assumes that everything in the organism remains the same, except for the mutated process and its direct derivatives, although it is to be expected that the organism will attempt to regain normalcy upon such a perturbation by evoking compensatory mechanisms. Thus, dynamic modeling is in principle more powerful but requires much more data support.

In the following, we describe case studies addressing lignin biosynthesis in different plants and with different methods. As stated before, we will focus primarily on data needs and different model uses.

### Models of lignin biosynthesis

#### Use of in vitro data

At present, metabolic modeling is far from having access to ideal comprehensive data obtained in vivo. To overcome this challenge, a common approach is the use of in vitro equivalents. An excellent example of this strategy in the context of lignin modeling is the work by Wang et al. [9], who constructed a dynamic model based on kinetic reaction and inhibition parameters of pathway enzymes in the black cottonwood, *Populus trichocarpa*. The authors derived 189 kinetic parameters associated with generalized Michaelis–Menten mechanisms, primarily in the form of *K*_cat_, *K*_*m*_, and *K*_*i*_ of the 21 enzymes involved in monolignol biosynthesis. They also measured absolute enzyme quantities using mass spectrometry. Furthermore, the authors used a measured S/G ratio to quantify the input flux with a customized optimization algorithm. Such optimization methods are often needed in large-scale metabolic modeling, because the number of fluxes is typically greater than the number of metabolites, which creates a mathematical situation that cannot be directly solved. The information from their experiments allowed Wang’s team to construct a fully parameterized model with estimated input flux, which they formulated as ordinary differential equations (ODEs). They were able to obtain the steady-state flux distribution and to investigate the effects of enzyme perturbations on lignin content and composition.

In principle, the well-established strategy used by Wang’s team is excellent, as it leads to a fully dynamic model that permits explanations and predictions. The somewhat disconcerting issue is the use of in vitro data, which at present seems unavoidable, but leads to the following questions: (1) To what extent are in vitro data accurate and representative of the pathway behavior in vivo, and does enough in vivo information exist to validate the results of such models? In other words, it is unclear how to assess the reliability of these models. (2) It is clear that no biomathematical modeling effort can presently claim to have taken all components and modulators of a pathway into account. Thus, is it possible to ensure that all relevant information is present quantitatively to reproduce and explain in vivo observations? Or is it simply not feasible to reconstruct the complex in vivo cell environment with sufficient reliability from in vitro information? For example, Wang et al. did not include the enzyme caffeoyl shikimate esterase [10] in their poplar lignin model [9]; this enzyme was discovered as a new component in the lignin pathway while their studies were ongoing.

These concerns are not exaggerated and can even be found in a very detailed microbial investigation by Teusink et al. [11], which provides a good perspective in this regard based on the much simpler pathway system of glycolysis in baker’s yeast, *Saccharomyces cerevisiae*. Specifically, these authors compared in vivo flux and concentration profiles with the results of a computational model that had been constructed based on the best available kinetic parameters obtained in vitro. Despite the authors’ dedicated efforts to use the same yeast source and obtain measurements under the same assay conditions, the discrepancies between the model results and the observed in vivo behavior were alarming. For possible explanations, Teusink et al. pointed to potential factors that may be active in vivo and cause uncertainties that are almost impossible to implement in in vitro models. Some of these uncertainties are apparently not adjustable by tuning of rate constants or through modifications in the model structure, but may be due to complicated combinations of molecular interactions between the pathway metabolites and enzymes or agents outside the investigated metabolic pathway. The authors proffered that these small details might have caused drastic differences during the integration of in vivo information into systemic models.

Similar concerns about in vitro–in vivo extrapolations were voiced some while ago by Savageau and others [12, 13], while Albe and Wright and others came to the conclusion that such an extrapolation is, by and large, justified in many cases [14, 15]. In any case, these problems are disconcerting, as in vivo measurements are incomparably more difficult to perform than experiments in vitro. Then again, if it is only possible to obtain in vitro data, are there means of in vivo validation? It appears that a direct validation of individual process representations will be difficult. Thus, one must hope that different types of in vivo data may fill the gap as they are combined with models designed from in vitro data for more reliable results.

#### Use of limited in vivo data

##### Lignin synthesis in poplar

At present, the total body of in vivo data is dwarfed by information obtained in vitro, and this situation is not likely to change any time soon. As a case in point here, the lignin pathway simply does not permit many concentration or flux measurements in vivo. Instead, the typical dataset that can reasonably be expected today consists of lignin content and composition under different conditions, possibly augmented with a few metabolite concentrations. Although this situation might seem to be quite dire for modeling, mathematical and computational approaches can still offer interesting results.

As a pertinent example, Lee and Voit [16] investigated the lignin biosynthesis pathway in *Populus* xylem based on a relatively small set of data consisting of the S/G ratios and down-regulation levels of enzymes in five transgenic plants. In addition, the authors used information regarding the pathway stoichiometry, regulatory information of five enzymes of the pathway, and an enzyme capacity measurement for COMT. Utilizing a predetermined lignin monomer composition as input and maximum lignin production as the cell’s alleged objective, the authors were able to generate steady-state flux distributions through FBA methods. Furthermore, to convert this information into a dynamic model, they employed a strategy derived from biochemical systems theory (BST) [17–21]. In this modeling framework, all fluxes are represented with power-law functions, so that the parameters can be coarsely estimated without knowledge of direct measurements.

Once the model was fully parameterized, the authors were able to run simulations that ultimately reproduced the lignin composition in all measured transgenics. In fact, an entire ensemble of models was generated, rather than a single model with a unique set of parameter values. This ensemble of models was validated against two transgenics that had not been used to set up the model. Upon validation, an indirect optimization method [22] was implemented to propose enzyme profiles that were expected to lead to a minimal S/G ratio in order to minimize recalcitrance. Single, double, and triple enzyme alterations were conducted to give insights and to determine the most effective perturbations. An interesting detail to note is that the best triple mutation did not contain the double mutation plus an additional mutation, but a different set. Specifically, in comparison to the wild-type S/G ratio of about 1.8, the model predicted a minimal S/G ratio of about 1.3 for two modifications, namely reduction of COMT and CAld5H activities, but a minimum of about 1.1 for three modifications in which the activities of C4H, CAD, and CAld5H were somewhat increased. These computational predictions have not been tested in actual plants.

The fact that a model is able to predict the results of perturbations is intriguing, especially because the power-law representation does not explicitly model specific reaction mechanisms, but only the overall effect of a metabolite or regulator on a given process. Then again, the in vivo data used to formulate and instantiate the model encapsulate in some sense everything occurring in the plant, which is not the case for in vitro models. Encouragingly, the estimated parameters in Lee’s analysis are in agreement with biochemical knowledge of the pathway and provide new insights into the dynamics of the pathway (see results in [16]). Similarly, the predictive capacity of the model to characterize the best candidates for gene alterations is interesting, but it remains to be seen whether explanations and hypotheses obtained with the model are comparable with those obtained with a model like Wang’s [9], which was based on experimentally laborious in vitro data.

##### Lignin synthesis in alfalfa

The structure of the lignin biosynthesis pathway and its regulation in alfalfa (*Medicago sativa* L.) are fairly well known, but some observations on transgenics were confusing as they seemed to contradict the pathway structure. In particular, some gene knock-downs led to different S/G ratios even though they occurred before the branch point where the pathways toward S- and G-monolignols diverge. Lee et al. [8] set out to investigate this situation, using an in vivo dataset of lignin content and composition in eight stem internodes in wild-type and seven transgenic lines (with reduced PAL, C4H, HCT, C3H, CCoAOMT, F5H, or COMT activity). The internode classification in this case provided the opportunity to characterize the differential biosynthesis of lignin during the maturation of stem tissue.

Without formal computation, an analysis of the logic of the pathway topology mandated the reversibility of the enzymatic steps catalyzed by HCT and C3H (Fig. 1), which had not been considered before. Taking this reversibility into account did not resolve the puzzle regarding S/G ratios though. Thus, the authors constructed a computational model of the pathway by first using FBA to compute the steady-state flux distribution in wild type, and then applying the method of MOMA [7] to analyze the redistribution of fluxes in transgenics. This analysis revealed that the results regarding S/G ratios in transgenics could not be explained unless functional channels were active to partition the pathway flux into dedicated S- and G-pathways.

Using statistical analysis, the authors showed that there was a strong correlation between the flux catalyzed by CCR1 and the flux of the consecutive reaction catalyzed by CAD in all strains except for the CCoAOMT-deficient line. This curious result indicated a lack of product exchange between coniferyl aldehyde produced by either COMT or by CCR1. To examine this situation more carefully, the authors tested the possibility of kinetic regulation by the CCR2-COMT and CCoAOMT-CCR1 routes (Fig. 1), but extensive Monte-Carlo simulations indicated only a very remote possibility of kinetic regulation by substrate/product interactions. Instead, the analysis suggested regulation by one or more distant metabolites. The authors proposed that salicylic acid (SA) could act as the potential regulator of the pathway leading to S-lignin synthesis. Indeed, experimental data characterizing the correlation between SA and lignin content supported the computational hypothesis. Moreover, additional in vivo data, demonstrating the co-localization of COMT and F5H [23, 24], provided further evidence supporting the channeling hypothesis.

Wang et al. [9] criticized Lee’s approach on grounds that the method was rather indirect and, in particular, suggested that a complete kinetic model would be able to capture the experimental data without the need for channeling. While the existence of channels awaits further validation with direct experimental means, it is unclear whether a bottom-up kinetic approach would have led to the crisply targeted hypothesis of differentially regulated channels directing flux toward either S- or G-lignin.

**Fig. 2:**
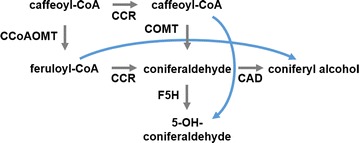
Metabolic channeling in *Medicago* proposed by Lee et al. [25]. The two crossing channels are associated with coniferaldehyde (see Fig. 1)

In a different study, Lee et al. [25] investigated the channeling hypothesis in *Medicago* by setting up an ensemble of dynamic kinetic models in 19 pathway configuration variants. Each of these variants preserved mass conservation, while allowing alternative routes including one or two metabolic channels across coniferaldehyde (Fig. 2). The models also examined the presence or absence of putative regulatory mechanisms. Extensive Monte-Carlo simulations over a biologically meaningful range of kinetic values identified only 6 among the 19 plausible configurations as feasible and demonstrated that only 4 out of 16 combinations of plausible regulatory mechanisms could match the experimental data. A graph analysis of these six configurations showed that they were topologically closely related and corresponded to a closed network, if closeness between two configurations was defined as a difference in only one enzymatic reaction. Interestingly, all six feasible configurations in the analysis included one or both proposed metabolic channels.

While the computational results strongly suggest the existence of channels, and independent experimental evidence supports these results [8, 23, 24], it is of course imaginable that other explanations could be found for the counterintuitive data in alfalfa, because even the best model fit to data can never offer a guarantee that the model is in some sense correct or that there could not be other models satisfying the same data in a similar manner. It is interesting though, that the computational results were inferred directly from actual data from these same species and with a minimum of assumptions, whereas models based on in vitro data, obtained from bacteria, should be validated in the target species in situ, before they can be considered true. Furthermore, while the power-law formulation used by Lee is mathematically guaranteed to be correct at an operating point of choice, there is no such guarantee for Michaelis–Menten functions; in fact, it is clear that their underlying assumptions and prerequisites are violated in situ [2, 12, 26].

##### Lignin synthesis in switchgrass

Similar to the investigations on poplar and alfalfa, a limited dataset characterizing lignin content and composition was available for switchgrass (*Panicum virgatum*) [27], one of the most promising plants in bioenergy research. This dataset was used to set up a model of lignin biosynthesis and to examine for this species the hypothesis of channeling at a diverging branch point, leading to either S- or G-lignin. Specifically, wild-type and four transgenic (4CL, CCR, CAD, and COMT) lignin profiles were analyzed with FBA methods to compute steady-state flux distributions. The stoichiometric model included three variants permitting alternative, slightly differing pathways with and without a hypothetical metabolic channel comprising CCR and CAD. Extensive Monte-Carlo simulations generated thousands of random kinetic parameters to test whether any of the three configurations could reproduce the experimental data in a dynamical manner. Surprisingly, none of the configurations was able to capture the increase in H-lignin in 4CL-transgenics. Instead, the computational results suggested the necessity to include product inhibition by downstream pathway metabolites, as well as substrate competition between CCR substrates. These computational suggestions identified *p*-coumaroyl-CoA and feruloyl-CoA as possible regulators that were arguably necessary to reproduce the observed increases in H-lignin. The model also revealed that the reaction catalyzed by 4CL, which converts ferulic acid into feruloyl-CoA, constitutes an impediment for explaining the counterintuitive accumulation of ferulic acid in COMT transgenics.

Further computational analysis suggested the accumulation of some so-far unidentified metabolite as an inhibitor of 4CL and as the mechanism by which ferulic acid increased. Revisiting the experimental data indicated a slight accumulation of *p*-coumaric acid and caffeic acid, which was shown to suffice to support the model-based hypothesis. Taken together, the pathway configuration including both the CCR-CAD channel and two independent CCR and CAD reactions, along with the deduced regulatory mechanisms, turned out to be the only structure capable of matching the in vivo data. The authors validated the model to some degree by testing the responses to an enzyme expression profile in an independent transgenic PvMYB4 line that had not been used at all to set up the model. Overall, the analysis produced satisfactory results with respect to lignin content and composition, as well as the concentration profiles of several of the pathway intermediates [27, 28].

#### Use of pathway data and ^13^C-labeling data in *Brachypodium distachyon*

This case study describes new results that have not been published so far. For this reason, a short description of methods is provided in a later section.

**Fig. 3:**
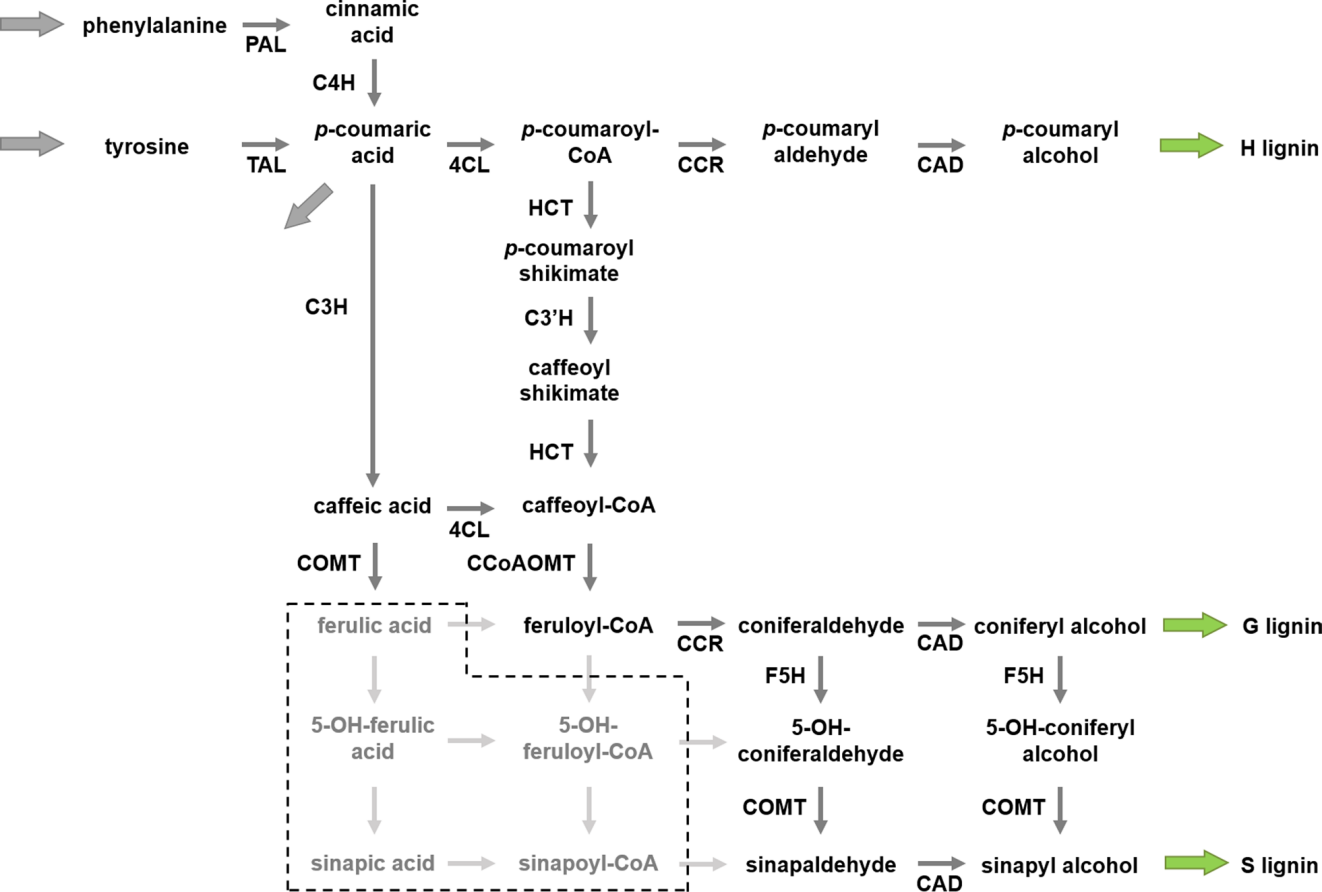
Putative lignin biosynthesis pathway in *Brachypodium distachyon*. *Brachypodium* can use both phenylalanine and tyrosine as substrates for lignin biosynthesis. At this point, the direct conversion of *p*-coumaric acid into caffeic acid and the existence of C3H in this organism are speculative. Reactions shown in the shaded box have not been fully explored in the current literature

While the results of analyzing in vivo alfalfa and switchgrass transgenics data in a somewhat indirect manner were interesting and could be validated to some degree, the data themselves constitute a rather thin base for model development. This base becomes more solid if it is combined with other types of data. An example for such a merging of heterogeneous data types is the lignin biosynthetic pathway in *Brachypodium distachyon*. In contrast to dicots, monocot grasses use both phenylalanine and tyrosine as the initial substrate for monolignol production (Fig. 3). One puzzling aspect of this apparent redundancy is that, despite the nearly equal contribution of both precursors to the total lignin content, phenylalanine is preferentially incorporated into G-lignin, and tyrosine into S-lignin, although both pathways converge at the same intermediate metabolite, *p*-coumaric acid [29]. This result is surprising and cannot easily be explained with putative structure of the lignin pathway in *Brachypodium*. Beyond the existence of this intermediate, where the two pathways converge, the G- and S-lignin pathways appear to be the same until they split at the coniferaldehyde node.

A computational model directly corresponding to the alleged pathway structure (Fig. 3) confirms the logic-based analysis: the pathway, as currently alleged, cannot reproduce key observations, such as the differential channeling of phenylalanine and tyrosine toward G- and S-lignin. Specifically, model simulations demonstrate that the pathway scheme in Fig. 3 is unable simultaneously to satisfy the following observed requirements:

- Match the amount of ^13^C-labeled H-lignin in experiments with [U-^13^C_9_]phenylalanine;
- Match the observed ^13^C incorporation into ER-bound ferulic acid in the same experiment;
- Capture the differential ^13^C incorporation levels from [U-^13^C_9_]phenylalanine and [U-^13^C_9_]tyrosine in lignin units.

One great advantage of a modeling approach is the relative ease with which it is possible to test different hypotheses and variations of the pathway structure in order to obtain possible explanations. As a specific example, it was reported that the three enzymes C4H, C3′H and F5H of the lignin biosynthesis pathway in *B. distachyon* are bound to the outer surface of the ER, while the remaining enzymes are located freely in the cytosol ([29]; unpubl. data). This finding led to the hypothesis that the spatial localization of enzymes might be a reason for the preferential incorporation of phenylalanine and tyrosine into different monolignols. This hypothesis was readily tested with a computational model that distinguishes the two locations (see below). These two locations, or compartments, are physically not strictly separated, but allow the handing over of metabolites through diffusion.

**Fig. 4:**
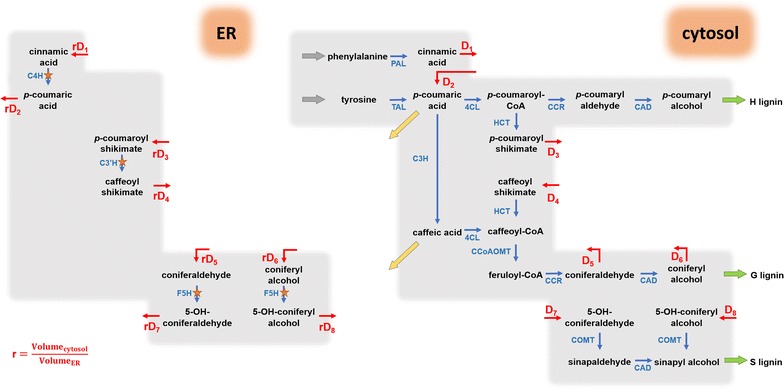
Proposed compartmentalized pathway of lignin biosynthesis in *B*. *distachyon*. The blue arrows represent enzymatic reactions within each compartment. The blue arrows marked by orange stars depict reactions whose catalytic enzymes are bound to the outer ER surface. The red arrows show diffusion fluxes between the compartments. The two yellow arrows are effluxes. The quantity *r* is a compensation constant to address the different volumes of the compartment

**Fig. 5:**
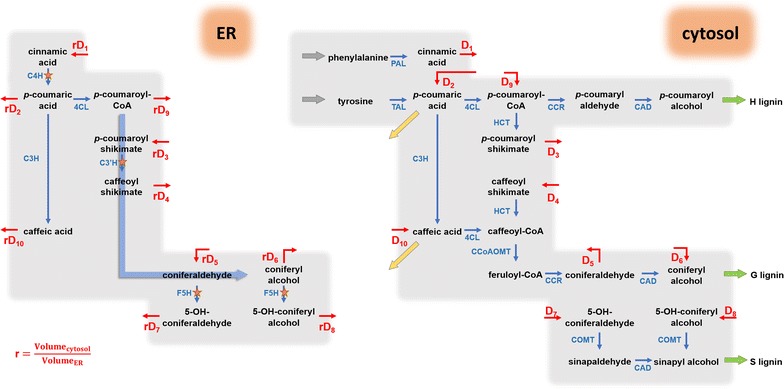
Extended compartmentalized lignin pathway model in *B*. *distachyon*. Conversion of *p*-coumaric acid to *p*-coumaroyl CoA by 4CL and diffusion flux *D*_9_ are necessary to explain label incorporation into H-lignin in experiments with labeled phenylalanine. Conversion of *p*-coumaric acid to caffeic acid by C3H and the diffusion flux *D*_10_ are necessary to explain label incorporation into ferulic acid in the same labeling experiments with phenylalanine. The metabolic channel in the ER compartment keeps some of the ^13^C-label from being diluted by the cytosol diffusion fluxes and permits preferential incorporation of phenylalanine and tyrosine in S- and G-lignin

To test the hypothesis of two distinct locations, we set up a refined model scheme by assigning the reactions catalyzed by the ER-bound enzymes, C4H, C3′H and F5H, to the ER compartment, and all others to the cytosol compartment (Fig. 4). While there is no strict spatial separation between ER and cytosol, we assumed preferential enzyme activity within each compartment and slower diffusion between compartments. As a note, only the net diffusion fluxes are shown in the pathway model, but both forward and reverse diffusions are considered explicitly in the computational model (see later section). Specifically, we took the following steps for our model design. In the current scheme (Fig. 4), the only means for incorporation of ^13^C into H-lignin is through the diffusion flux D_2_, and this flux is diluted with the influx from unlabeled tyrosine. To increase ^13^C incorporation into H-lignin, a second diffusion flux, D_9_, is added between the ER compartment downstream of D_2_, and this flux compensates for the dilution of tyrosine (Fig. 5). This diffusion flux D_9_ can be interpreted as partial activity of 4CL in the ER compartment.

Beyond the inconsistent amount of H-lignin, low incorporation of ^13^C in ferulic acid in the [U-^13^C_9_]phenylalanine labeling experiment is an indication for dilution by unlabeled tyrosine through caffeic acid. Therefore, a downstream influx, D_10_, from the ER compartment is postulated to compensate for tyrosine dilution and to increase ^13^C incorporation in wall-bound ferulic acid. Again, this flux corresponds to partial activity of C3H in the ER compartment (Fig. 5).

Closer inspection of the pathway reveals that the key site for preferential incorporation of [U-^13^C_9_]phenylalanine and [U-^13^C_9_]tyrosine into different lignin units is the branch point where the pathways toward G- and S-lignin diverge; this divergence happens at the coniferaldehyde node. The original scheme in Fig. 4 dictates the same level of ^13^C-labeling into both G and S units, due to dilution in both compartments at the coniferaldehyde node into the free cytosol. To explain the actually observed higher incorporation of ^13^C into G-lignin in the phenylalanine labeling experiment, an undiluted upstream flux from the ER is necessary to compensate for the dilution from the cytosol influx (D_5_ and D_6_) into the immediate G-lignin precursors coniferaldehyde and/or coniferyl alcohol. We first modeled this hypothesis by simply adding a suspected direct flux from *p*-coumaroyl-CoA into coniferyl alcohol (Fig. 5, thick blue arrow).

Simulations with this amended model showed that the scheme in Fig. 5 is able to capture the levels ^13^C incorporation in H-lignin and ferulic acid from [U-^13^C_9_]phenylalanine experiments. Also, by acting as a metabolic channel, the direct flux from *p*-coumaroyl-CoA into coniferyl alcohol shields the flow within the ER compartment from strong dilution by diffusion from the cytosol compartment, and thereby enables the preferential incorporation of phenylalanine and tyrosine–born carbons into different monolignols units.

**Fig. 6:**
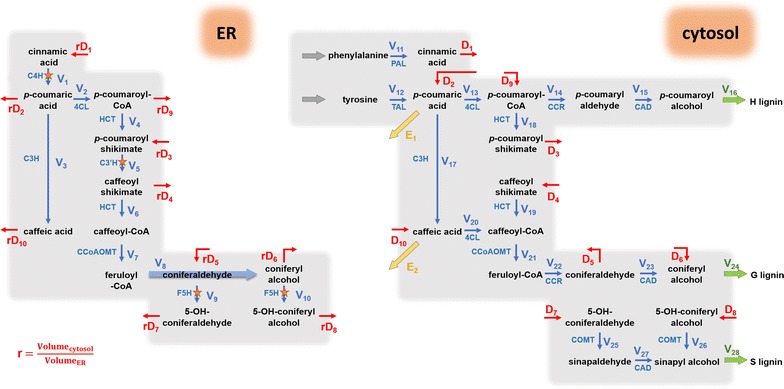
Revisited compartmental model of lignin pathway with the shortest feasible metabolic channel. The CCR/CAD channel (*V*_8_) appears to be the shortest path that is able to preserve the flow in the ER compartment from complete dilution by cytosol diffusion fluxes

While the long metabolic channel in Fig. 5 is able to simulate the preferential incorporation of precursors into lignin units, it is intriguing to determine whether fewer enzymes in such channel could still reproduce the data. Therefore, we examined the scheme in Fig. 5 toward the shortest channel possible (Fig. 6). This analysis suggested that the critical point to shield the ER compartment from strong dilution by cytosolic diffusion fluxes is coniferaldehyde. Without this compound protected, G- and S-lignins cannot attain different ^13^C_9_-labeling levels. If this conjecture can be validated, the simplest scheme consists merely of a CCR/CAD channel.

**Fig. 7:**
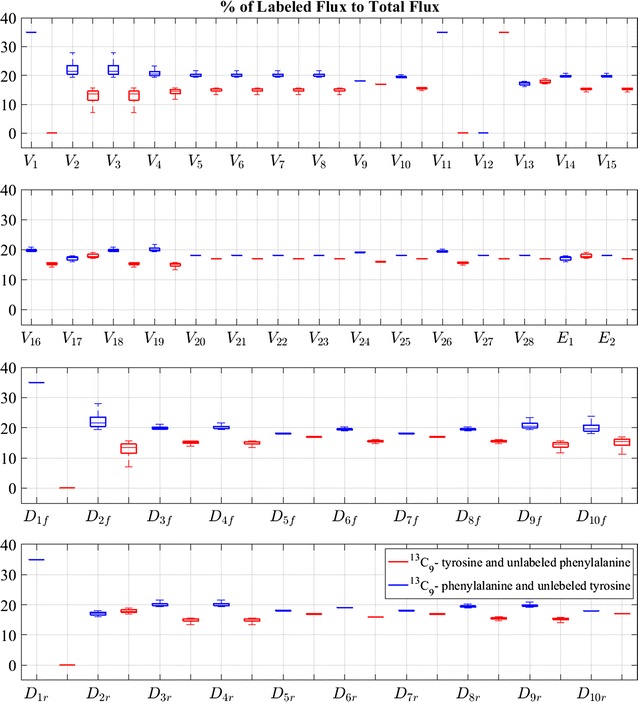
Steady-state flux distribution of labeled fluxes in *Brachypodium*. The results compare the percentage of steady-state labeled flow within the steady-state total flux in [U-^13^C_9_]phenylalanine and [U-^13^C_9_]tyrosine experiments; they correspond to the pathway scheme in Fig. 6. Both directions of diffusion for each diffusion flux are shown: *D*_*if*_ aligns with the direction of *D*_*i*_ in Fig. 6 and *D*_*ir*_ with the opposite direction (see Modeling ^13^C-labeling experiments section)

**Fig. 8:**
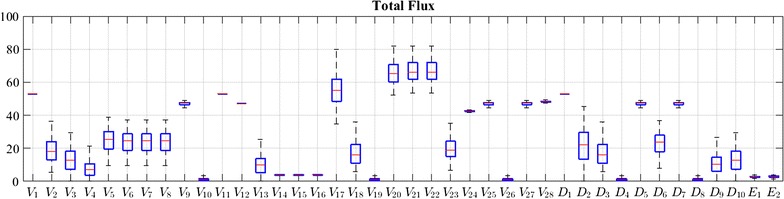
Total steady-state flux distribution in *Brachypodium*. The total flux includes both labeled and unlabeled fluxes. The results correspond to the scheme in Fig. 6

Simulations of the scheme in Fig. 6 resulted in steady-state flux distributions that capture the experimental ^13^C-labeling data (Fig. 7). Phenylalanine and tyrosine contribute nearly equally to the resulting lignin content: in the [U-^13^C]phenylalanine experiment, 35% of phenylalanine is labeled and tyrosine is unlabeled (natural abundance), while in the [U-^13^C_9_]tyrosine experiment, 35% of tyrosine is labeled and phenylalanine is unlabeled (natural abundance). The labeled fluxes in Fig. 7 compare the contributions of phenylalanine and tyrosine in each pathway flux. Figure 8 exhibits the total flux values, which combine the values of labeled and unlabeled fluxes. Since the magnitude of the input flux is unknown, we normalized the input to a base value of 100 units of mass per unit of time.

Because the system is mathematically underdetermined, its steady-state solution is not unique (see Steady-state analysis section). Therefore, a range of admissible steady-state values is possible for each flux. It is worth emphasizing in this context that all solutions in the resulting ensemble are consistent with all pertinent observations; namely:

- Each model in the ensemble captures the experimental data with respect to the label distribution in steady-state fluxes. For instance, *V*_24_ shows a higher labeled portion than *V*_28_ when phenylalanine contains the feeding label;
- The lignin compositions and S/G ratios in all scenarios are compatible with experimental data;
- The labeled lignin composition is compatible with ^13^C_9_-phenylalanine and ^13^C_9_-tyrosine experimental data; and
- The labeled ferulic acid and *p*-coumaric acid match with experimental data.

Further details are presented in Table 1.

**Table 1:** Computational model results compared to experimental data.The model results demonstrate a good match with experimental data in terms of total lignin (A) and the incorporation of label (B)

A				
	H/total lignin (%)	G/total lignin (%)	S/total lignin (%)	S/G
Experimental data	4	41	55	1.09
Model result	4.1	45	51	1.13

B						
	H-lignin^a^ (%)	G-lignin (%)	S-lignin (%)	Total lignin (%)	*p*-Coumaric acid (%)	Ferulic acid (%)
Label incorporation in [U-^13^C_9_]phenylalanine feeding experiment						
Experimental data	36	22.3	21	22.2	21	23
Model result	19.6	19.1	18.1	18.6	17.2	18
Label incorporation in [U-^13^C_9_]tyrosine feeding experiment						
Experimental data	24.6	16.5	18.1	18.6	17	13
Model result	15.4	15.9	16.9	16.4	17.8	17

^a^Label incorporation in H-lignin was not considered as a criterion during the model calibration. The recorded experimental value in the [U-^13^C_9_]phenylalanine feeding experiment is greater than the reported label level in phe, which is 35% [29]. As a consequence, we deemed the measurement unreliable and did not use labeled H-lignin measurements

The boxplots in Figs. 7 and 8 reflect the distributions of admissible values. As can be seen, *V*_8_ admits small values in comparison to its parallel reactions in cytosol compartment, i.e., *V*_22_ and *V*_23_. This result demonstrates that, while the main pathway for the reactions catalyzed by CCR and CAD resides in the cytosol, a relatively small and undisturbed flux through CCR/CAD at the ER is sufficient to establish the metabolic channel necessary for preferential incorporation. In fact, considering the wrinkled environment of the ER surface, it is not hard to imagine that localized pools would keep a small fraction of the pathway undisturbed from exchanges of metabolites with the cytosol.

## A brief review of modeling methods for pathway analysis

### Generic model formulation

In a kinetic systems model, the dynamics of the pathway is represented by a system of ordinary differential equations (ODEs) in which the metabolites are the states. The rate of change in each metabolite is determined by sums and differences of all fluxes that directly affect this metabolite. Each flux is a mathematical function of the metabolites and other variables of the system that needs to be selected. Although the fluxes are usually nonlinear functions, the collection of fluxes itself forms a linear system, which can be represented as a matrix equation of the type

1 $$\dot{X}\, = \,S \cdot V.$$

Here, *X* is the vector of metabolites, $\dot{X}$ is its derivative with respect to time, *S* is the stoichiometric matrix, and *V* is the vector of fluxes. The stoichiometric matrix *S* defines the pathway structure. An element *S*_*i*,*j*_ of this matrix equals 1 if flux *V*_*j*_ is directed toward metabolite *X*_*i*_. It is − 1, if flux *V*_*j*_ removes material from metabolite *X*_*i*_, and it is equal to 0, if flux *V*_*j*_ has no direct effect on metabolite *X*_*i*_. In long form, the matrix equation can be rewritten for each equation as

2 $$\dot{X_{i}}\, = \,\sum\limits_{j\, = \,1}^{n} {S_{i,j} V_{j} }$$

where *n* is the total number of fluxes.

### Steady-state analysis

The steady state of a system is important for two reasons. First, many biological systems tend to operate close to such a state, where the overall concentrations of metabolites do not change, even though flux is running through the system. Second, from a mathematical point of view, many analyses at a steady state are much simpler than for the differential equations themselves, because now one has, by definition, $\dot{X}\, = \,0$, so that all differential equations become explicit algebraic equations that can be analyzed with methods of linear algebra. If all fluxes are known, it is usually not difficult to compute the steady-state of a system. However, the reverse is not true: if only the metabolite concentrations at the steady state are known, it is not easy to compute the corresponding flux distribution, because metabolic systems almost always contain more reactions than variables. In this case, optimization methods like FBA or MOMA need to be employed.

In the *Brachypodium* study, we chose an alternative to FBA and MOMA. Namely, we intended to obtain the most likely solution without specifying an objective function for the FBA optimization. Because the degrees of freedom of a solution to our system are directly associated with diverging branch points, we focused on the flux split ratios (FSRs) at these points. In cases where these FSRs were known, we used their values; otherwise, we performed large-scale Monte-Carlo simulations with thousands of combinations of FSRs and retained only those solutions where all fluxes were positive at all time points of an experiment. This strategy led to the most likely flux profiles. Details of this method are discussed in [30].

**Fig. 9:**
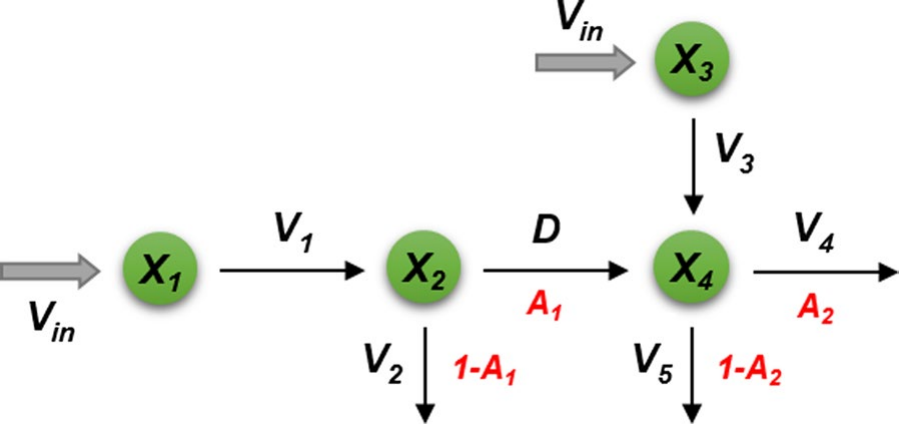
Material flow through net fluxes in an illustration example. Without labeling, it is sufficient to model diffusion fluxes as net fluxes. However, this is not the case for labeling experiments (Fig. 10)

As a simplified example, consider the hypothetical pathway in Fig. 9, which has two FSRs, and hence two degrees of freedom.

The system of differential equations corresponding to pathway in Fig. 9 is

3 $$\begin{aligned} &\dot{X}_{1} \, = \,V_{in} \, - \,V_{1} , \\ &\dot{X}_{2} \, = \,V_{1} \, - \,V_{2} \, - \,D, \\ & \dot{X}_{3} \, = \,V_{in} \, - \,V_{3} , \\ & \dot{X}_{4} \, = \,V_{3} \, + \,D\, - \,V_{4} \, - \,V_{5} . \end{aligned}$$

Given a set of metabolite concentrations over time, the pathway can be driven by infinitely many flux distributions [31]. To determine the most likely, the system in Eq. (3) is first rewritten in terms of FSRs of the system at the steady state ($\dot{X}\, = \,0$).

4 $$\begin{array}{ll} V_{1} = V_{in} , & \quad V_{3} = V_{in,} \\ V_{2} = \left( {1 - A_{1} } \right) \cdot V_{1} , & \quad V_{4} = A_{2} \cdot \left( {V_{3} + D} \right),\\ D = A_{1} \cdot V_{1} & \quad V_{5} = \left( {1 - A_{2} } \right) \cdot \left( {V_{3} + D} \right). \end{array}$$

Now, thousands of pairs (*A*_1_, *A*_2_) of FSRs are randomly generated by Monte-Carlo sampling with *A*_*i*_ ∊ [0, 1]. Each pair, entered into the model, yields steady-state values of the fluxes *V*_1_,…, *V*_5_ and *D*. These are filtered to retain only desired fluxes. For instance, in the actual case study of *Brachypodium*, only those flux profiles are retained that satisfy the following criteria:

- Fluxes take only non-negative values at all time points;
- The lignin composition and S/G ratio are compatible with experimental data.

It is theoretically possible that the estimation strategy based solely on split ratios does not converge to an acceptable solution, and we have discussed means of addressing this situation elsewhere [30]. Here the split-ratio method succeeded without the need for alternative methods.

### Modeling ^13^C-labeling experiments

**Fig. 10:**
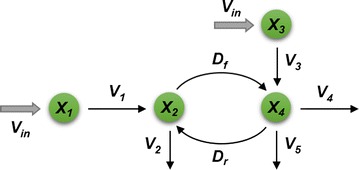
Illustration of the flow of label in the same example as Fig. 9, but with explicit flux directions. In contrast to the scenario in Fig. 9, labeling experiments mandate the modeling of diffusion fluxes in both directions. Specifically, the simpler model in Fig. 9 does not allow flow of label from *X*_4_ to *X*_2_ through *D* when labeled metabolite is fed to the pathway through *X*_3_, but the figure here demonstrates that such flow is clearly possible

The diffusion flux between two pools of the same metabolite in different locations is comprised of two directions (Fig. 10). Although the two opposing fluxes have a net value, as shown in Figs. 6 and 9, it is necessary to consider them individually when modeling a labeling experiment. The reason is that the labeling content of each pool affects the flow of label, but the net diffusion alone would not reflect the free passing of label in both directions. For instance, the illustration scheme in Fig. 9 does not allow flow of label from *X*_4_ to *X*_2_ through *D* when labeled metabolite is fed to the pathway through *X*_3_, but due to the bidirectional nature of diffusion fluxes, it is evident that flow would happen in reality. As Fig. 10 illustrates, these bidirectional diffusion fluxes form cycles and don’t allow the direct computation of steady-state fluxes.

To tackle this issue, we first consider only the net diffusion flux as shown in Fig. 9 and compute the steady states. Then we consider the bidirectional model in Fig. 10, employ Eq. (2), and use conservation of mass for labeled and total fluxes at each metabolite. For a given labeling percentage *L*_*i*_, where *L*_*i*_ represents the labeled portion of the pool of metabolite *X*_*i*_, we obtain for the pathway system in Fig. 10:

5 $$\begin{aligned}& V_{1} + D_{r} = V_{2} + D_{f} , \\ & L_{1} V_{1} + L_{4} D_{r} = L_{2} V_{2} + L_{2} D_{f} , \end{aligned}$$

which can be rewritten as

6 $$\begin{aligned} & D_{r} = \frac{{\left( {L_{2} - L_{1} } \right)V_{1} }}{{L_{4} - L_{2} }}, \\ & D_{f} = \frac{{\left( {L_{4} - L_{1} } \right)V_{1} }}{{L_{4} - L_{2} }} - V_{2} . \end{aligned}$$

Similar calculations for pool *X*_4_ yield

7 $$\begin{aligned} & V_{3} + D_{f} = V_{4} + V_{5} + D_{r} , \\ & L_{3} V_{3} + L_{2} D_{f} = L_{4} V_{4} + L_{4} V_{5} + L_{4} D_{r} , \\ & D_{r} = \frac{{\left( {L_{2} - L_{3} } \right)V_{3} }}{{L_{2} - L_{4} }} - V_{4} - V_{5} , \\ & D_{f} = \frac{{\left( {L_{4} - L_{3} } \right)V_{3} }}{{L_{2} - L_{4} }}. \end{aligned}$$

By equating *D*_*f*_ from Eqs. (6) and (7) one obtains

8 $$L_{4} = \frac{{L_{2} V_{2} - L_{1} V_{1} - L_{3} V_{3} }}{{V_{2} - V_{1} - V_{3} }}.$$

Assuming that *L*_1_ and *L*_3_ are known from inputs of the pathway, *V*_*in*_, we can compute *L*_4_ from the computed steady-state fluxes in the previous step and an estimated *L*_2_. Therefore, *L*_2_ is the only unknown to be estimated, and *L*_4_, *D*_*f*_ and *D*_*r*_ can consequently be computed. In fact, we only need to estimate the label level of one of the parallel metabolite pools in the cytosol and ER compartments.

Similar to the use of split ratios, vector *L* is generated randomly by Monte-Carlo sampling, and the labeled fluxes can then be computed. The labeled fluxes corresponding to Fig. 10 are

9 $$\begin{array}{*{20}c} \begin{aligned} V_{1,L} = L_{1} \cdot V_{1} , \hfill \\ V_{2,L} = L_{2} \cdot V_{2} , \hfill \\ V_{3,L} = L_{3} \cdot V_{3} , \hfill \\ V_{4,L} = L_{4} \cdot V_{4} , \hfill \\ V_{5,L} = L_{4} \cdot V_{5} , \hfill \\ D_{f,L} = L_{2} \cdot D_{f} , \hfill \\ D_{r,L} = L_{4} \cdot D_{r} , \hfill \\ \end{aligned} & {} & \begin{aligned} V_{1,U} = \left( {1 - L_{1} } \right) \cdot V_{1} , \hfill \\ V_{2,L} = \left( {1 - L_{2} } \right) \cdot V_{2} , \hfill \\ V_{3,U} = \left( {1 - L_{3} } \right) \cdot V_{3} , \hfill \\ V_{4,U} = \left( {1 - L_{4} } \right) \cdot V_{4} , \hfill \\ V_{5,U} = \left( {1 - L_{4} } \right) \cdot V_{5} , \hfill \\ D_{f,U} = \left( {1 - L_{2} } \right) \cdot D_{f} , \hfill \\ D_{r,U} = \left( {1 - L_{4} } \right) \cdot D_{r} . \hfill \\ \end{aligned} \\ \end{array}$$

Closer inspection demonstrates that the model in Fig. 9, which considers only net diffusion, computes the labeled portion of *D* as *L*_2_*D*, which is equal to *L*_2_(*D*_*f*_ − *D*_*r*_), whereas Eq. (9) quantifies the net labeled flux as *L*_2_*D*_*f*_ − *L*_4_*D*_*r*_.

The fluxes for the *Brachypodium* example were defined in this manner. Labeled fluxes that satisfied the model criteria for labeling experiments were recorded. The criteria were

- The labeled lignin composition is compatible with ^13^C_9_-phenylalanine and ^13^C_9_-tyrosine experimental data; and
- The labeled ferulic acid and *p*-coumaric acid levels match the experimental data.

The recorded flux vectors were plotted using boxplots, which offer a visual representation of the distribution of most likely flux values within their admissible ranges.

## Discussion

Mathematical modeling in biology is still in its infancy. Especially within the realm of plant and crop science, the number of modeling articles is negligible in comparison to experimental papers. As a consequence, the collective experience with plant and crop modeling approaches is still limited, and much more practice and many more case studies will be needed to gain a glimpse into the systemic responses of plants to interventions and manipulations. It may even be, as some experts claim (Leroy Hood, *pers. comm.*), that a “new math” is needed that allows us to combine different data and heterogeneous information in a more efficacious manner than is possible today. Ultimately, a deeper understanding of such responses would allow us to answer questions like “how does ‘a’ plant react to natural or artificial changes?” or “why does plant (or plant species) A respond differently to a perturbation than plant (or plant species) B?”

To obtain more practice and experience of this type, experimentalists and modelers should collaborate more closely. On the one hand, modelers will need experiments specifically performed for some modeling aspects. At present, many data are available, and the data flow from -omics experiments can be overwhelming. However, not all data are useful for the type of modeling outlined in this article, and modelers will be dependent on experimentalists to perform other types of experiments [32]. On the other hand, experimentalists will want to see genuinely new results coming out of models, especially if they had contributed data to the modeling effort. They will benefit from new, integrative interpretations of their data and from reliable modeling results and computationally achieved hypotheses guiding the “next steps” in their research programs. The generic differences between laboratory or field experiments and computational approaches render it evident that this type of collaboration has true potential, but that it will take time and patience on both sides to make progress toward reaching some of this potential.

As a tangible target, experimentalists and modelers should explore together to what degree metabolic responses can be predicted (qualitatively or quantitatively) from the existence of genes and enzymes (as, for instance, TAL in the case of *Brachypodium*) or from quantitative transcriptomics, where one would expect to find similarities between gene expression and changes in enzyme activities, which however do not always materialize in reality, due to post-transcriptional alterations. It would also benefit both sides to obtain and computationally analyze data describing the same process in different species, as we demonstrated here with the different models for lignin biosynthesis. At first, these comparative analyses could shed light on questions such as: whether apparent differences between species are experimental or modeling errors; whether different designs have evolved in line with the general phylogeny of these species or whether they are due to other factors; and whether different natural designs are dictated by different environmental needs or demands. Together, these combined analyses would have the potential of revealing design principles that govern these processes and could provide deep explanations for why certain species solve a task in the observed fashion and not in a different fashion.

As an example, we discussed lignin biosynthesis. The lignin heteropolymer and its monomers are quite similar among different species and, indeed, the intermediates of their biosynthetic pathway are essentially the same. However, the enzymatic reactions of the pathway exhibit striking differences, not just in terms of their kinetic features or parameters, but even in their existence. These differences raise the question why one pathway design is favored in one case, but not in another. The answer to this question is not only academically interesting but is of immediate pertinence to the biofuel scientist and metabolic engineering in general, because ignorance of the true reasons mandates a new conceptual model for every untested species. By contrast, knowledge of a general design or operating principle would allow predictions regarding the pathway topology of a new species based on the criteria on which the principle is founded.

The different pathway models for lignin synthesis in a number of plant species [8, 9, 16, 25, 27, 28] have revealed commonality, but also differences in regulatory features and, as suggested here, compartmentalization. One could thus come to the conclusion that every species manages its lignin production differently. However, the fact that some distinctive features of one model are not part of the other models should not be over-interpreted, at least not quite yet. It is well possible that the pathway in alfalfa and switchgrass is as compartmentalized as the one in *Brachypodium*, but the dictum of simplicity in modeling, and the data that these models were based upon, suggested that specific compartments were not needed to match the data in these species. The same is true for other apparently distinguishing features, such as product inhibition, which we found necessary in switchgrass, and which may well be in effect in other species. These features were needed to make the models consistent with specific data typesets, and if one re-analyzed the models with other types of data types, the same features could well be suggested for other species. As it stands, the collective experimental database is sparse, and the published models are minimalistic special cases of the same “master model,” which can even account for the fact that some species seem to be missing certain pathway metabolites or enzymatic reactions. It remains to be the subject of further data generation and analysis to determine whether these differences disappear toward one common, complex model, whether they are immaterial byproducts of evolution that did not exert strong selective pressure, or whether they evolved for reasons that are germane to these species and their environments. The cooperation between experimentalists and modelers has led to early successes. Some of these are narrowly focused by explaining observations that had been puzzling before. We described some of these in the context of lignin synthesis and recalcitrance. To study some of the in vivo complexity in an in vitro system, the lignifying cell suspension cultures reported in several species (*Arabidopsis* [33], poplar [34], switchgrass [35]) could be useful for modeling purposes of the lignin pathway. These systems can be studied along a time course when lignin deposition and cell differentiation occur, allowing the evaluation of different parameters such as pH or temperature and the use of dynamic models that could be proposed as potential in vivo validation systems.

Others studies have attempted to connect several scales of the biological hierarchy of processes, both in time and size (e.g., [36–41]). Not surprisingly, such much larger models cannot account for every detail at every lower level. Nonetheless, it might be useful, for instance, to use agent-based models at the highest scale considered, such as overall plant growth, and to anchor into them detailed models of key sub-systems, such as photosynthesis, respiration, and stress responses. Instead of an agent-based model, the highest level could also be a dynamic FBA model [42], or it is even possible to use low-level models as constraints in genome-wide metabolic models [43].

The epitome of such models is whole-plant-plus-environment models that have been developed in recent years and capture governing processes and responses quite well. For instance, SOYSIM [44] is a computational model that simulates soybean growth on a daily basis throughout its lifecycle. It permits reasonably accurate explorations of water use, additional irrigation, and potential yield under different conditions. Another example is the WIMOVAC simulation model, which allows investigations of the carbon balance in plants and permits predictions of crop responses to changes in climate [45–48]. It is applicable to different plant and soil types and can be used by researchers, managers and students as an exploratory tool. These organismal models are now to be coupled more comprehensively to environmental and agricultural models, an effort that has recently been coined *Crops* in silico [49–51]. To be successful, this effort will require the close collaboration not only between experimental plant scientists and mathematical modelers, but also involve experts in biophysics, hydrogeochemistry, meteorology, high-performance computing, visualization, and many other fields. The challenges are grand indeed, but a solid foundation is being built by the collaboration of several communities, such as BESC [52], and it will only be a matter of time and collective willpower to increase momentum allowing us to achieve some of the set goals.
